# The Main Theories on the Pathogenesis of Endometriosis

**DOI:** 10.3390/ijms24054254

**Published:** 2023-02-21

**Authors:** Jelizaveta Lamceva, Romans Uljanovs, Ilze Strumfa

**Affiliations:** 1 Faculty of Medicine, Riga Stradins University, 16 Dzirciema Street, LV-1007 Riga, Latvia; 2 Department of Pathology, Riga Stradins University, 16 Dzirciema Street, LV-1007 Riga, Latvia

**Keywords:** endometriosis, pathogenesis, immune regulation, oestrogen, progesterone, stem cells, metaplasia, epigenetics, carcinogenesis

## Abstract

Endometriosis is a complex disease, which is defined by abnormal growth of endometrial tissue outside the uterus. It affects about 10% of women of reproductive age all over the world. Endometriosis causes symptoms that notably worsen patient’s well-being—such as severe pelvic pain, dysfunction of the organs of pelvic cavity, infertility and secondary mental issues. The diagnosis of endometriosis is quite often delayed because of nonspecific manifestations. Since the disease was defined, several different pathogenetic pathways have been considered, including retrograde menstruation, benign metastasis, immune dysregulation, coelomic metaplasia, hormonal disbalance, involvement of stem cells and alterations in epigenetic regulation, but the true pathogenesis of endometriosis remains poorly understood. The knowledge of the exact mechanism of the origin and progression of this disease is significant for the appropriate treatment. Therefore, this review reports the main pathogenetic theories of endometriosis based on current studies.

## 1. Introduction

### 1.1. Epidemiology

Endometriosis is a chronic gynaecological condition, which is characterized by abnormal presence of endometrial glands and stroma outside the uterus accompanied by chronic inflammation. Most commonly it affects organs of the pelvic cavity: ovaries, fallopian tubes, urinary bladder, intestines or peritoneum [1,2]. Rarely, it is localized in other organs outside the pelvis—diaphragm, pleura, abdominal wall, central or peripheral nervous system [2]. Endometriosis is mainly found in girls and women of reproductive age. According to the World Health Organization data, there are approximately 10% of reproductive-aged women (190 million) globally who are diagnosed with this condition [1]. The peak age of patients is in the time frame between 25 and 45 years [3]. Usually it takes up to 8 to 10 years to reach the diagnosis of this disease [4]. Endometriosis has a considerable impact on worldwide economics as well—it costs the world over 80 billion USD per year [5].

### 1.2. Symptomatology

Endometriosis has variable range of manifestations—from accidentally found asymptomatic lesions to severe condition, which does not depend on the size of the lesion [3]. Most often the first symptoms show up before the age of 20 [3]. The main symptoms caused by endometriosis are chronic pelvic pain, severely painful menstrual periods, dyspareunia, dysuria and/or painful defecation, abdominal bloating and constipation. It may also increase the risk of mental health issues, such as anxiety and depression. The other manifestation of endometriosis is infertility without any other symptoms: 40–50% of infertile women are diagnosed with endometriosis [2,5]. There are different mechanisms how endometriosis can influence fertility: distorted anatomy of the pelvic cavity, development of the adhesions, fibrosis of the fallopian tubes, local inflammation of the pelvic structures, systemic and local (i.e., endometrial) immune dysregulation, changes in hormonal environment inside the uterus and/or impaired implantation of the embryo [3]. In addition, the disease has a significant negative impact on quality of life and social well-being of patients—due to pain and other symptoms, e.g., fatigue, severe bleeding or mood swings, women have to skip their studies or work and might tend to avoid sex. 

### 1.3. Subtypes of Endometriosis

The main classification of endometriosis is based on its localization and histopathology; there are three subtypes: superficial peritoneal endometriosis, ovarian endometriotic cysts and deep infiltrating endometriosis. Superficial peritoneal endometriosis rarely causes severe clinical symptoms. It is found on the surface of organs of the pelvic cavity and often attaches to the peritoneum. Ovarian endometriotic cysts appear on the ovaries and form cystic structures known as endometriomas or “chocolate cysts”. These cysts are filled with fluid and vary in size. This subtype is associated with infertility and ovarian cancer. Deep infiltrating endometriosis can invade visceral organs to a depth of 5 mm or more within or outside the pelvic cavity and distort local anatomy. It is a rare form of endometriosis, and the cause of significant symptoms, so it requires surgical treatment frequently [6].

The pathogenesis of endometriosis still has many questionable aspects, that is why it is a relevant topic for the research. Nowadays, there are many theories and evidence for each of them. The full understanding of the mechanism of this condition would help to develop the most effective treatment, which is still limited now.

In the present article we summarized the main theories on the pathogenesis of endometriosis according to the relevant research data.

## 2. Theories on the Pathogenesis of Endometriosis

### 2.1. Retrograde Menstruation

The theory about retrograde menstruation is well-known as Sampson’s theory. It remains relevant since it was described for the first time in 1925. The main idea of it is that menstrual blood containing endometrial cells regurgitate via patent fallopian tubes into the peritoneal cavity, where the implantation of these cells might occur [7,8]. After implantation, development and growth of the lesion is supported by angiogenesis [9]. It is possible because of activated peritoneal macrophages, which produce angiogenic factors, e.g., vascular endothelial growth factor (VEGF) [10].

The problem of this theory is that retrograde menstruation might explain ovarian and superficial peritoneal endometriosis, but not deep infiltrating endometriosis or lesions outside the peritoneal cavity [6,7,8,9].

However, several studies show that reflux of menstrual blood is physiological for women with patent fallopian tubes, and most of them (76–90%) experience retrograde menstruation without further endometriosis development [9,11]. The cases, when endometriosis develops in women, who have retrograde menstruation, could be explained by epidemiological studies which expose the risk factors of endometriosis—short menstrual cycle, longer menstrual flow and uterine outflow obstruction. These factors increase the quantity of retrogradely flushed cells [11,12].

Researching this theory, the baboon model has been used for some studies, because of ethical reasons on women’s examination. Serial diagnostic laparoscopies have been performed to evaluate the amount and composition of menstrual blood in the peritoneal cavity in different phases of menstrual cycle. Results on experiments with baboons show the correlation between retrograde menstruation and the development of endometriosis, but researchers admit that most likely this is not the single one pathogenetic reason for the disease [11].

### 2.2. Benign Metastasis

In 1927 Sampson suggested one more pathogenetic mechanism—theory of metastatic endometriosis. This theory assumes that a small amount of the endometrial tissue can be disseminated through the uterine-draining lymph vessels during menstruation. This theory is based on his finding: there was an endometrial polyp projecting into the lumen of a lymph vessel [13]. The benefit of a benign metastasis theory is that this pathogenetic mechanism can explain the occurrence of endometriosis in lymphatic nodes and distant locations such as lungs, because lymphatic capillaries are found in almost all organs [14].

Nowadays, there are some reports of lymph node endometriosis, confirmed by histopathological examination that shows the presence of endometrial glandular and stromal cells in lymph node, and immunohistochemistry that is positive for oestrogen receptor, progesterone receptor, PAX8 and CD10 [14,15,16,17].

Research on lymphangiogenesis has discovered that there is a dysregulation of the expression of lymphangiogenic growth factors and their receptors in the eutopic endometrium of ladies diagnosed with endometriosis. The main promoters of lymphangiogenesis in endometrium are VEGF-C and VEGF-D [14,18,19], which are upregulated by proinflammatory cytokines interleukin 1β (IL-1β), tumour necrosis factor α (TNFα), IL-7 and CD74 [14]. In addition, the density of lymphatic microvessels of eutopic endometrium of patients is increased, too. So, these changes together could facilitate the entry of endometrial tissue into the lymphatic circulation [14,19]. However, it is still unclear how this dysregulation actually affects the development of endometriosis. 

### 2.3. Immune Dysregulation

**Figure 1:**
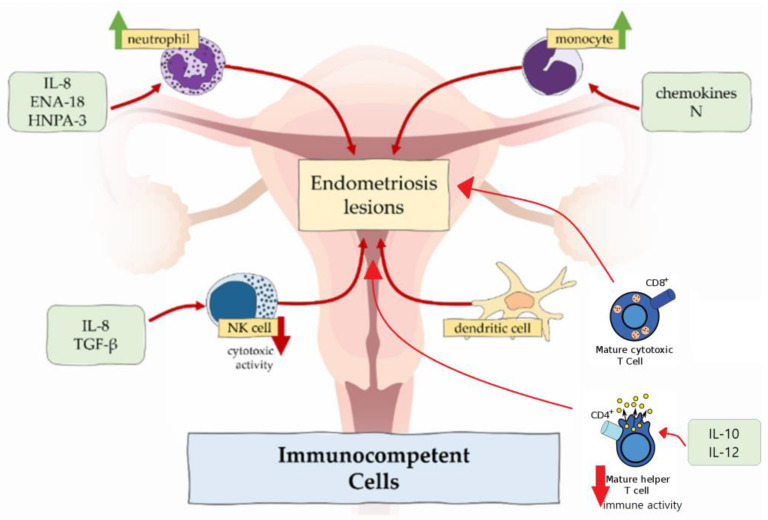
Immunocompetent cells in endometriosis. Figure replicated from [21] under Creative Commons license, provided at https://creativecommons.org/licenses/by-nc/4.0/ (accessed on 17 January 2023). Changes made: Figure legend, figure modification.

Inflammation, caused by immune dysregulation, is one of the main mechanisms that takes part in diseases where cell proliferation and infiltration occur. In case of endometriosis, proinflammatory pathways block functions of apoptotic mechanisms, and potentially harmful cells adhere to distant sites [20]. Immune cells involved in formation and further development of endometrial lesions are macrophages, neutrophils, NK cells, dendritic cells and T cells (Figure 1). 

#### 2.3.1. Macrophages

Macrophages detect and phagocytose pathogens and foreign cells, act as antigen-presenting cells to activate T cells and participate in tissue regeneration of healthy endometrium [20,21,22]. Normally, macrophages represent approximately 10% of total immune cell population in the proliferative phase in endometrium. They change in number according to the menstrual cycle phase, regulated by oestradiol and progesterone. During menses, their number is significantly increased in accordance with their phagocytic function—clearing apoptotic cells and cell debris during endometrial shedding [22].

In endometriosis, number of macrophages is increased in eutopic endometrium [21,22] and peritoneal fluid [10] across all phases of menstrual cycle and without cyclic changes [23]. In contrast, phagocyte function is decreased because of the reduced expression of CD3, CD36 and annexin A2 [10,21,24]. It results in incomplete endometrial shedding, presence and survival of desquamated tissue in the peritoneal cavity [21]. Peritoneal macrophages release proinflammatory cytokines TNFα, IL-6, IL-8, IL-1β, which recruit neutrophils, provoke inflammation and support the development of endometrial lesions [3,10]. Macrophages also produce VEGF, which promotes angiogenesis in endometriosis [10].

Moreover, some studies found the predominance of M2 macrophage subtype in endometriotic lesions and peritoneal cavity [10,25]. This subtype classically promotes development of the tumours, e.g., colorectal cancer and osteosarcoma. It means that endometriosis shares some characteristics, e.g., inflammation and tissue invasion, with neoplastic processes, but is still classified as a benign disease. In addition, M2 promotes nerve fibre growth, so predominance of this subtype of macrophages could be related to severe pain, experienced in women with endometriosis [21,22]. 

#### 2.3.2. Neutrophils

In healthy endometrium, neutrophils are involved in endometrial repair and regulation of cyclic vascular proliferation. 

In women with endometriosis, neutrophil counts in peritoneal fluid are increased. This could be attributable to the locally increased concentration of chemoattractants secreted by epithelial cells such as IL-8, epithelial neutrophil-activating peptide 8 (ENA-78) and human neutrophil peptides 1-3 (HNP1-3), which attract neutrophils to the peritoneal cavity [10,21].

According to the results of the mouse model study, depleting of the neutrophils with anti-Gr-1 antibody in the early stage of endometriosis significantly decreased the number of endometrial lesions [26]. In contrast, this antibody had no effect in advanced disease, which suggests that neutrophils do not take part in endometriosis progression, but only in induction [10]. However, neutrophils express cytokines, e.g., VEGF, IL-8, C-X-C chemokine motif ligand 10 (CXCL10), which cause progression of the disease [10].

#### 2.3.3. NK Cells

Role of the NK cells in the immune system is the following: they produce cytokines, which control tumour immunity and microbial infections. Regarding endometriosis, their cytotoxic function is suppressed by the IL-6, IL-15 and transforming growth factor β (TGF-β) [10,27]. Therefore, endometrial cells, which enter the peritoneal cavity, tend to stay there. However, the amount of the NK cells shows no differences in women with and without endometriosis.

#### 2.3.4. Dendritic Cells

Dendritic cells are responsible for antigen presentation to T cells and, therefore, are involved in immune responses in mucosal surfaces [22]. There are two types of dendritic cells—plasmocytoid dendritic cells and myeloid dendritic cells. Plasmocytoid dendritic cells are involved in recognition of viruses and produce interferons, while myeloid dendritic cells are involved in T cell activation and represent the most relevant cells to endometriosis. In healthy individuals, the amount of the dendritic cells increases to clear endometrial debris during menstruation. In women affected by endometriosis, there is significantly reduced density of myeloid dendritic cells in endometrium [28]. In the peritoneal cavity, numbers of dendritic cells are increased and may promote neuroangiogenesis, causing and enhancing pain sensation [22].

#### 2.3.5. T Cells

One of the important factors, which maintains the development of endometriosis, is imbalance between type 1 T lymphocytes (Th1) and type 2 T lymphocytes (Th2). These two types have different functions in the immune system: Th1 lymphocytes produce cytokines and promote cellular responses, but Th2 lymphocytes influence differentiation of B lymphocytes and suppress cellular and humoral responses [3]. In endometriosis, Th2 lymphocytes represent the main population of T cells, so potentially harmful cells stay unrecognized. On the other hand, the immune response of CD4+ Th1 lymphocytes in peritoneal fluid is suppressed due to an increased expression of IL-10 and IL-12 [29]. 

Moreover, the peripheral concentration of cytotoxic (CD8+) T cells and activated (HLA-DR) T cells in healthy women increases in luteal phase compared with the follicular phase of the menstrual cycle, but there are no such fluctuations of cytotoxic and activated T cells in patients with endometriosis [30].

Recently, the association between regulatory T cells (Treg cells) and endometriosis was reported. The main function of regulatory T cells is modulation of the immune system, maintaining tolerance to self-antigens and preventing autoimmune diseases [29]. In endometriosis patients, there is an increased amount of Tregs in the peritoneal fluid and decreased—in the peripheral blood. These changes can lead to the development of autoimmune reactions and suppress local cellular immune response [29].

### 2.4. Coelomic Metaplasia

In 1924 Robert Meyer proposed coelomic metaplasia theory. It is based on the female reproductive tract development: it develops from a pair of Müllerian ducts, which arise from coelomic epithelial cells of mesodermal origin [31]. This theory assumes that the original coelomic membrane undergoes metaplasia and forms endometrial stroma and glands. It is the most suitable explanation for cases of endometriosis in men, who have received high doses of oestrogen for prostatic carcinoma treatment, and Rokitansky-Kuster-Hauser syndrome patients who does not have functioning endometrial tissue because of congenital aplasia of the uterus and the upper part of the vagina [9,31,32]. In both these clinical groups, endometriosis cannot be explained by Sampson’s implantation theory due to lack of eutopic endometrium.

The most common form of endometriosis, which could be explained by this theory is ovarian endometrioma. The mesothelium, which derives from the coelomic epithelium covering the ovary, has great metaplastic potential and can invaginate into the ovarian cortex [9,32]. These mesothelial inclusions could be transformed into endometriosis by metaplasia [9]. Growth factors, which influence this phenomenon, are still unknown.

### 2.5. Embryonic Rest Theory

Embryonic rest theory is a sort of a metaplasia theory. It states that remnants of embryonic cells of Wolffian or Müllerian duct origin may differentiate into endometriotic lesions [12,33]. In the coelomic metaplasia theory, the transformation occurs only to mesothelium, but there is no such restriction in the embryonic rest theory [33].

In this theory, it is supposed that some changes in cell differentiation or relocation of the Müllerian ducts during embryogenesis of the fetus can maintain the spreading of embryonic cells—primordial endometrial cells [34]. Generally, these cells are located in the posterior pelvic floor and remain inactive until puberty, and then the process of formation of endometriotic lesions starts with oestrogen stimulation [12].

Recently, as a proof of this theory, Signorile et al. published their results on autopsy of female fetuses, where they found the presence of ectopic endometrium in the posterior pelvic floor structures: Douglas pouch, recto-vaginal septum, rectal tube and posterior wall of uterus [12]. These places are pretty common for diagnosed endometriosis cases.

This theory is suitable not only for endometriosis cases in women, but men too, because Wolffian ducts also contain embryonic cells.

### 2.6. Endometrial Stem Cell Recruitment Theory

Stem cells represent a minor fraction of multipotent cells with high replicative potential, having unlimited ability to renew themselves and capability to produce more differentiated daughter cells [35].

Many studies have targeted the impact of stem cells on endometriosis in recent years. They show that there are several populations of somatic stem cells in endometrium, including epithelial, mesenchymal and mixed side population [6,36]. The main functions of these cells are remodelling, regeneration and homeostasis of the tissue. Epithelial stem cells are found in the basal layer, and are responsible for regeneration of the functional layer during the proliferative phase, but mesenchymal stem cells are localized in the perivascular area of the basal and functional layers and are responsible for generation of functional stroma [6,12].

The migration of endometrial stem cells remains hypothetical. Some of the above-listed theories could be used to explain the mechanism. Firstly, endometrial stem cells are also found in menstrual blood [36]. This blood containing stem cells can reach the peritoneal cavity via patient fallopian tubes as Sampson’s retrograde menstruation theory considers [12]. The second theoretical mechanism of the migration of stem cells to the ectopic sites is abnormal cell migration during organogenesis of the female reproductive tract, which is associated with aberrant expression of WNT and HOX genes [36]. The last mechanism is an ability of endometrial origin stem cells to enter the angiolymphatic space passively during menstruation and move around the circulation [6].

After the migration phase, stem cells adhere and start to form endometrial lesion. The stem cell potential of lesion formation has been proven by Cervelló et al. in 2011, when endometrial side population cells were implanted beneath the kidney capsule in immunocompromised NOD-SCID mice and this experiment resulted in endometriosis [37].

This theory is an important finding because it can explain the pathogenesis of all three subtypes of endometriosis and its ectopic localization outside the abdominal cavity [6]. 

### 2.7. Bone Marrow-Derived Stem Cell Theory

This variant of stem cell recruitment theory is based on another source of stem cells—bone marrow. These cells are able to incorporate themselves into the endometrium to regenerate the tissue [36]. Several populations of cells take part in endometrial regeneration—mesenchymal, hematopoietic and endothelial progenitor cells [6].

The conception of theory is the following: bone marrow stem cells, which circulate via blood vessels, are settled in soft tissue instead of going to the endometrium, while reduced number of cells is recruited to eutopic endometrium [6,12]. Recent studies suggest that the CXCL12/CXCR4 axis is involved in recruiting bone marrow-derived stem cells, so the malfunction of this axis can cause the misplacement of stem cells [6]. 

The advantage of bone marrow-derived stem cell theory is its capability to explain extrapelvic endometriosis without the concept of “benign metastasis”.

### 2.8. Hormonal Imbalance

**Figure 2:**
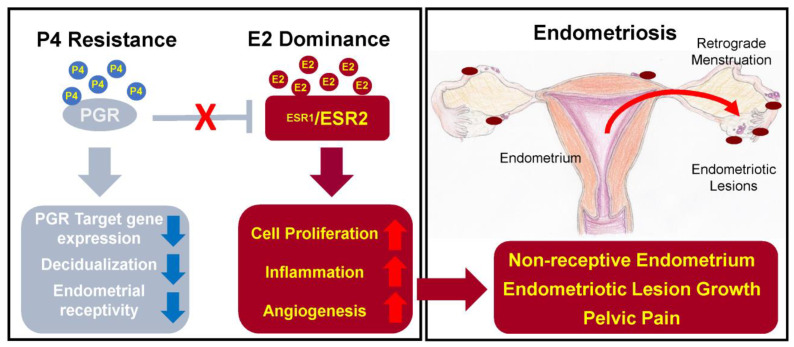
The effects of progesterone and oestrogen dysregulation on endometrium. Figure replicated from [38] under Creative Commons license, provided at https://creativecommons.org/licenses/by-nc/4.0/ (accessed on 17 January 2023). Changes made: Figure legend.

In the healthy endometrium, progesterone and oestrogen signalling is strictly coordinated and menstrual cycle phase-dependent. This is important to maintain a normal menstrual cycle, embryo implantation and development of the pregnancy [38]. Oestrogen induces epithelial proliferation during the proliferative phase, while progesterone inhibits the action of oestrogen and initiates the secretory phase, when stromal cells begin the decidualization [20,38]. The dysregulation of these two hormones—resistance to progesterone and oestrogen dominance [38,39]—leads to endometriosis development (Figure 2).

#### 2.8.1. Oestrogen

Endometriosis is often called an “oestrogen-dependant” disease. The reason for this statement is simple—endometriosis mostly affects women of reproductive age, but it also appears in women in postmenopausal age if the lady has high oestrogen level or undergoes oestrogen-replacement therapy [40].

The main functions of oestrogen in healthy endometrium include stimulation of epithelial proliferation and induction of leukaemia inhibitory factor (LIF), an IL-6 family cytokine, which is important for successful embryo implantation and decidualization of the endometrium [38].

In endometriosis, studies report higher levels of oestradiol—oestrogen steroid hormone—in menstrual blood and abnormal expression of enzymes involved in oestrogen metabolism, which can lead to increased oestrogen concentration and suppressed inactivation of oestrogen synthesis [40]. 

There are two oestrogen receptors—ERα and ERβ, which are coded by different genes: ESR1 and ESR2, respectively [38,39]. ERα and ERβ normally work together, but in endometriosis patients, expression of the receptors is changed—the ERα:ERβ ratio is significantly reduced due to high ERβ levels [41]. The main problem caused by abnormal expression of ERα is increased synthesis of inflammatory cytokines, prostaglandins, tumour-promoting and angiogenic factors [20,29]. On the other hand, ERβ overexpression leads to inhibition of TNFα-induced apoptosis and also promotes the inflammation [27,41]. As a result, synthesized prostaglandins induce inflammation and prevent cell apoptosis; tumour-promoting and angiogenic factors support the progression of the endometrial lesions and inhibition of apoptosis promotes cell proliferation and lesion growth [20,27]. 

In addition, oestrogen is able to stimulate growth of peripheral nerve fibres by upregulating nerve growth factors (NGF) causing nociceptive pain [27].

#### 2.8.2. Progesterone

The expression of the progesterone receptor (PGR) is induced by oestrogen action through its receptor ERα. PGR has two isoforms: PR-A and PR-B, expression of which increase during the proliferative phase and decrease after the ovulation [41]. Expressed PGR inhibits ERα expression, establishing a feedback system.

In endometriosis, as a result of low ERα:ERβ ratio and high oestrogen levels, progesterone resistance develops: PR-B is undetectable and PR-A levels are significantly lower than in the endometrium of healthy individuals [41]. Progesterone resistance manifests as a decreased responsiveness to progesterone of endometrial stromal cells [2].

Moreover, mutation of PGR causes sterility in mice due to reduced or absent ovulation, uterine hyperplasia, lack of decidualization of the endometrium and limited mammary gland development [38].

Therefore, to compensate the lack of progesterone, progestin therapy is one of the options of hormonal therapy for endometriosis. This therapy reduces endometriosis-related pelvic pain and eliminates laparoscopically visible endometrial lesions [41].

Due to the significant impact of oestrogen and progesterone on endometriosis development, the treatment mainly aims at the balance of these hormones. Therapy options that are currently in use include combined oral contraceptives, progestins, gonadotropin-releasing hormone agonists, danazol and aromatase inhibitors. The other options of treatment are still under development, e.g., gonadotropin-releasing hormone antagonists, selective oestrogen receptor modulators and selective progesterone receptor modulators [3,38]. However, hormonal therapy is associated with systemic adverse effects including weight gain, fluid retention, acne, hot flashes, decreased libido, insomnia and vaginal dryness [3], which might decrease the compliance to long-term hormonal treatment. 

### 2.9. Alterations in Epigenetic Regulation

In recent years, growing body of evidence suggests that epigenetic changes have a certain role in the development of endometriosis. Epigenetic changes are alterations of gene expression without any changes in DNA sequence. They are represented by the alterations in DNA methylation, histone acetylation, RNA transcription, chromatin remodelling, etc. [42]. The epigenome can be influenced by environmental factors, e.g., social behaviour, metabolism and nutritional deficiencies [39]. 

#### 2.9.1. DNA Methyltransferases 

The enzymes DNA methyltransferases are responsible for DNA methylation. Normally, the expression of DNA methyltransferases in endometrium is regulated by oestrogen and progesterone and varies depending on the cycle phase [42]. These enzymes are important for decidualization of the endometrium [42]. In endometriosis patients, hypermethylation of DNA of the local cells occurs due to increased expression of DNA methyltransferases DNMT1, DNMT3A and DNMT3B [39,43].

#### 2.9.2. Methylation of the HOXA10

Changes in methylation of the Human Homeobox A10 (HOXA10) genes are important because the dysregulation in some of these genes can lead to endometriosis.

HOXA10 expression is regulated by oestrogen and progesterone [39]. These genes are important for the endometrial changes throughout the normal menstrual cycle—they regulate endometrial growth, differentiation, and embryo implantation [39,44]. 

In patients affected by endometriosis, the expression of HOXA10 is decreased during the secretory phase, and, as the result, uterine receptivity is decreased and endometriosis-related infertility occurs [39,43]. Probably, HOXA10 gene expression is reduced due to hypermethylation of the HOXA10 gene promoter in the endometrial tissue [39,43].

#### 2.9.3. Histone Acetylation

The enzymes called histone deacetylases are responsible for histone modulation and acetylation. In endometriosis, activity of histone deacetylases HDAC1 and HDAC2 is increased. It leads to the hypoacetylation of cyclins, which causes cell cycle induction and propagation [42,43].

### 2.10. Micro-RNAs

Micro-RNAs are short non-coding RNA molecules that regulate translation of post-transcriptional mRNA by repression and mRNA degradation, acting as large-scale molecular switches [45]. According to recent findings, endometriosis is characterised by abnormal spectrum of micro-RNAs, further influencing the expression of the relevant target mRNAs [45]. Wide spectrum of micro-RNAs are involved in different steps of endometriosis. For example, miRNA-135a/b, regulating HOXA10, is upregulated in endometriosis and cause progesterone resistance [42,46]. MiR-199 is downregulated, so COX-2 translation is not suppressed, and it leads to pro-inflammatory prostaglandin synthesis such as IL-8 [42,45]. MiRNA-96b is also downregulated, and it is the cause of increased proliferation of the endometrial lesions [42]. MiR-126 increases VEGF and fibroblast growth factor (FGF) signalling in endothelial cells, resulting in neoangiogenesis and the development of a mature vasculature [45].

MiRNA-223 also showed a significant impact on endometriosis. This micro-RNA is involved in signal transduction, regulation of transcription, cell growth and development, modulation of inflammation and tumorogenesis [47]. In 2022, Xue et al. found that miRNA-223 is decreased in eutopic and ectopic endometrial stromal cells in women with endometriosis [47]. They also have proved that in case of upregulation of miRNA-223 proliferation, invasion and migration of endometrial stromal cells could be suppressed and epithelial-to-mesenchymal transition could be reversed as well [47]. These findings give miRNA-223 a potential of new therapeutic target.

The other micro-RNA, which promotes the growth, proliferation and angiogenesis of ectopic stromal cells, is miRNA-21 [46,48]. In the research by Wu et al. are presented three micro-RNAs with significant impact on endometriosis development. Expression of miR-26b-5p and miR-215-5p was downregulated, but miR-6795-3p (Table 1)—upregulated in serum of the patients, and their expression was stage related [48]. In addition, it has been found, that these three micro-RNAs are involved in such signalling pathways as MAPK and PI3K-Akt. MAPK signalling pathway is important in regulation of inflammation and the following cell processes: differentiation, division, proliferation, stress response, metabolism and apoptosis [48]. PI3K-Akt signalling pathway is also involved in cell processes and angiogenesis. These two pathways are significant for endometriosis development and further progression, that is why they could be used as therapeutic target too.

### 2.11. Carcinogenetic Pathways in Endometriosis

Despite the fact that endometriosis is classified as a benign disease, it still has a potential to transform into malignancy. This transformation is rather rare—it develops in 1% of all endometriosis patients [39,45]. Endometriosis-related malignant transformation most frequently affects ovaries, and the most common types of the ovarian malignancy are ovarian endometrioid carcinoma and ovarian clear cell carcinoma, which are found in 76% of all cases [39,49].

Recently, several carcinogenetic pathways have been reported for endometriosis-related malignant transformation. It is supposed that uncontrolled cell division, infiltration of surrounding tissues, neoangiogenesis and escape of apoptosis might be caused by the demethylation of oncogenes and the hypermethylation of tumour suppressor genes [39]. For example, the following events are involved in malignant transformation of the endometriosis: the hypermethylation of the human mutL homolog 1 (hMLH1) gene promoter which causes a decrease in DNA mismatch repair gene expression, the hypomethylation of long interspersed element-1 (LINE-1), inactivation of the tumour suppressor genes runt-related transcription factor 3 (RUNX3) gene and Ras-association domain family member 2 (RASSF2) gene by their promoter hypermethylation [39]. Also, in case of endometrioid cancer, there has been found an impact of activation of the KRAS oncogene and inactivation of the PTEN tumour suppressor gene [44,49]. Loss of PTEN activity is supposed to be an early event in malignant transformation of endometriosis, and is related to the mutation of PTEN gene itself [45]. In addition, Anglesio et al. found that in deep infiltrating endometriosis there are somatic mutations in cancer driver genes ARID1A, PIK3CA, KRAS, and PPP2R1A [50].

### 2.12. External Environmental Factors

Factors of the environment definitely have an impact on risks of endometriosis development. External factors can cause crucial changes in women organism under certain circumstances. But this section still has lack of evidence.

#### 2.12.1. Lifestyle

The main factors of the lifestyle that can provoke dysregulation in normal functioning of the organism are: lack of physical activity, smoking, caffeine and alcohol intake, diet.

It is supposed that physical activity helps to reduce the risk of endometriosis, because it decrease menstrual flow and normalize oestrogen balance [46]. 

Tobacco smoking increase the expression of pro-inflammatory mediators, disrupts synthesis of prostaglandin E2 and natural steroids [46]. Disrupted steroidogenesis leads to increased oestrogen and decreased progesterone synthesis [51].

Caffeine reduces production of Sex-Hormone Binding Globulin, it decrease the amount of bio-available testosterone, and as a result the levels of oestrogen reduce too [46]. It is suggested that caffeine has a protective potential due these changes, but the results of researches are still conflicting. On the other hand, Kechagias et al. found a correlation between endometriosis and caffeine intake in high quantities (>300 mg/day), admitting that it could be the risk factor for the disease [52].

Alcohol has the opposite effect on synthesis of hormones compared to caffeine. It has an impact on pituitary luteinizing hormone and activates the enzyme aromatase, resulting in increased oestrogen production and increased testosterone conversion to oestrogen, but it depends on certain dose of the alcohol [46,51].

The main dietary factor which could negatively affect on endometriosis development is increased red meat consumption, because of content of saturated fat [51,53]. 

#### 2.12.2. Dioxins and Polychlorinated Biphenyls (PCBs)

Dioxins and polychlorinated biphenyls (PCBs) are organic pollutants produced by industrial processes. The most toxic environmental pollutant is called 2,3,7,8-tetrachlorodibenzo-p-dioxin (TCDD) [53]. PCB and TCDD have an ability to disrupt an endocrine system. In context of endometriosis, it was found that increased amount of PCB and TCDD have been accumulated in adipose tissue of patient with deep infiltrating endometriosis [51,54], but the mechanism of this changes remains uncertain.

## 3. Conclusions

Endometriosis is a chronic disease which affects a significant number of women all around the world. Their quality of life is strongly reduced by disturbing symptoms and negative emotional experience which is associated with general health status, diagnosis and adverse effects of the treatment. 

It is clear that the pathogenesis of endometriosis is complex and involves many factors and processes which occur simultaneously. There are multiple interactions of the immune system, hormones, genes, local and stem cells—everything has an impact on endometriosis development and its further progression. In recent years, many theories have been studied, but there is no single theory which could explain all aspects of endometriosis. The future concept of endometriosis is likely to incorporate the elements from all the listed pathogenetic theories.

## Figures and Tables

**Table 1:** Micro-RNAs and their effects.

Micro-RNAs	Changes	Effect
miRNA-135a/b	Upregulated	Dysregulation of HOXA10 expression, progesterone resistance
miR-199	Downregulated	Synthesis of pro-inflammatory prostaglandins due to lack of COX-2 suppression
miRNA-96b	Downregulated	Increased proliferation of the endometrial lesions
miR-126	Upregulated	Neoangiogenesis due to increased VEGF and FGF
miRNA-223	Downregulated	Proliferation, invasion, migration of endometrial stromal cells, epithelial-to-mesenchymal transition
miRNA-21	Upregulated	Growth, proliferation and angiogenesis of ectopic stromal cells
miR-26b-5p	Downregulated	Activation of MAPK and PI3K-Akt pathways: inflammation, cell growth, differentiation and proliferation, angiogenesis
miR-215-5p	Downregulated	
miR-6795-3p	Upregulated	

## Data Availability

Not applicable.

## Abbreviations

CXCL10	C-X-C chemokine motif ligand 10
ENA-78	Epithelial neutrophil-activating peptide 8
ER	Estrogen receptor
FGF	Fibroblast growth factor
hMLH1	Human mutL homolog 1
HNP1-3	Human neutrophil peptides 1-3
HOXA10	Human Homeobox A10
IL	Interleukin
KRAS	Kirsten rat sarcoma virus
LIF	Leukaemia inhibitory factor
LINE-1	Long interspersed element-1
NGF	Nerve growth factors
NK cells	Natural killer cells
NOD-SCID	Nonobese diabetic/severe combined immunodeficiency
PCBs	Polychlorinated biphenyls
PGR	Progesterone receptor
PTEN	Phosphatase and tensin homolog
RASSF2	Ras-association domain family member 2
RUNX3	Runt-related transcription factor 3
TCDD	2,3,7,8-tetrachlorodibenzo-p-dioxin
TGF-β	Transforming growth factor β
Th1 and Th2	Type 1 T lymphocytes and type 2 T lymphocytes
TNFα	Tumour necrosis factor α
Treg	Regulatory T cells
VEGF	Vascular endothelial growth factor
